# Molecular Mechanism of NLRP3 Inflammasome in Inflammatory Diseases and Tumors

**DOI:** 10.1002/iid3.70213

**Published:** 2025-07-16

**Authors:** Le‐Lan Gong, He‐Jiang Zhou, Quan‐Ming Zhao, Ning Xu, Feng‐Chang Huang, Ling‐Yan Su, Wen‐Liang Li

**Affiliations:** ^1^ The Third Affiliated Hospital of Kunming Medical University Kunming China; ^2^ College of Food Science and Technology Yunnan Agricultural University Kunming China; ^3^ Yunnan Provincial Laboratory of Precision Nutrition and Personalized Manufacturing Yunnan Agricultural University Kunming China; ^4^ Department of Oncology The First Affiliated Hospital of Kunming Medical University Kunming China

**Keywords:** inflammatory diseases, metabolism disease, NLRP3 inflammasome, tumors

## Abstract

The nucleotide‐binding and oligomerization domain‐like receptors (NLRs) are pattern recognition receptors. Nucleotide‐binding oligomerization domain‐like receptor family pyrin domain‐containing 3 (NLRP3), a member of the NLRs family, can form a protein complex with caspase‐1, apoptosis‐associated speck‐like protein and caspase recruitment domain. And its assembly and activation cause inflammatory reaction and are closely related to the effects of antitumor immunity. The activation of NLRP3 inflammasome can induce polarization, hyperactivity or pyroptosis of immune cells, releasing interleukin‐1β (IL‐1β) and interleukin‐18, which leads a cascade immunity or inflammatory responses. As an important component of the innate immune system, the NLRP3 inflammasome plays vital roles inflammatory diseases and tumors. In this review, we attempt to summarize the recent findings about the role of NLRP3 in the pathogenesis of tumors and inflammatory diseases such as diabetes, Alzheimer disease, and atherosclerosis.

## Graphical abstract

My review paper described the struction of NLRP3 inflammasomes and the actived mechanism of them. Then, sumerized the NLRP3 inflammasomes' function of inflammatory diseases and cancer.

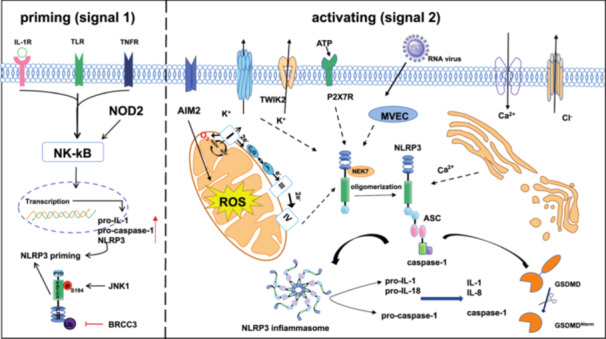

## 1 Introduction

The innate immune system is the first line of defense against pathogen's insults and recognize defective cell to initiate a protective response [1]. The system consists of tissue barrier (skin and mucosal system, blood‐brain barrier, placenta barrier, etc.); innate immune cells (phagocytes, killer cells, dendritic cells, etc.); and innate immune molecules (complements, cytokines, enzymes, etc.) [2]. Among them, phagocytes recognize danger‐associated molecular patterns (DAMPs) and pathogen‐associated molecular patterns (PAMPs) through germline‐encoded pathogen recognition receptors (PRRs) to mobilize inflammatory response, but PRRs strictly distinguish between their own and microbial components to prevent the development of autoimmune diseases [3]. The PRRs have been identified with six different genetic and functional branches: Toll‐like receptor (TLR), C‐type lectin receptor (CLR), retinoic acid‐inducible gene‐I‐like receptor (RLR), oligomerization domain‐like receptors (NLRs), cyclic AMP‐GMP synthase and stimulator of interferon genes, and absent in melanoma 2‐like receptors [4, 5]. TLRs and CLRs are transmembrane receptors while nucleotide‐binding NLRs and RLRs are cytoplasmic receptors [6]. There is increasing data suggesting that NLR is a modulator of TLR, RLR, and CLR signaling pathways. NLR recognizes ligands from a variety of sources which are subdivided into NLRP and NLRC based on their domain structure. Several inflammasomes have been described, including NLRP1 (mouse NLRP1b), NLRP3, and NLRC4 [7].

Inflammasomes are multimeric cytoplasmic protein complexes assembling in response to DAMPs and PAMPs which trigger the release of the pro‐inflammatory cytokines IL‐1β and IL‐18 to activate an inflammatory response. NLRP3 inflammasome respond to cellular perturbations and various microorganisms.

NLRP3 is activated upon stimulation by sterile triggers or microorganisms. Activated NLRP3 recruits a dimeric protein called apoptosis‐associated spot‐like protein, which contains the caspase activation and recruitment apoptosis‐associated speck‐like protein (ASC) [8] to participate in caspase‐1 activation. In addition, caspase‐dependent Gasdermin D (GSDMD) is also recruited into the NLRP3 complex and releases its N‐terminal structural domain, mediating membrane pore formation [9] and inflammatory cytokines, such as IL‐1β. Therefore, activation of NLRP3 inflammasome is essential for host defense against pathogen invasion and maintenance of homeostasis in vivo [10]. However, excessive activation of NLRP3 inflammasome can also lead to a hyper‐inflammatory state, ultimately leading to auto‐inflammatory disease, neurodegenerative disease, and cancer progression [11]. In recent years, we had more an understanding of the mechanistic connection between inflammatory diseases and cancer, but there remain many clinical questions [12].

In this review, we will summarize the studies regarding the molecular mechanism of the activation of NLRP3 inflammasome and its role in inflammatory diseases and tumors, which will promote understanding of the NLRP3 role in inflammatory diseases and tumors.

## 2 NLRP3 Inflammasome

NLR family proteins share a common feature, all centrally composed of a central nucleoside triphosphatase domain (NACTH), C‐terminal leucine‐rich repeats (LRRs), and N‐terminal effector domains, which can divide NLR into four subfamilies: NOD‐like receptor family acidic transcriptional structural domain (NLRA), NOD‐like receptor family baculovirus repressive repeat structural domain (NLRB), NOD‐like receptor family caspase activation and recruitment structural domain (NLRC), and pyridine (PYD) structural domain (NLRP) [10]. NLRP research are more notable. Most NLRP act as PRRs by recognizing PAMPs such as bacterial, muramyl dipeptide, RNA and DNA, viral, fungal, and protozoan, or DAMPs such as ATP, cholesterol crystals, calcium pyrophosphate dihydrate crystals, hyaluronic acid as well as environmentally derived irritants (silica, UV radiation) as ligands to activate inflammatory responses [11]. What's more, the NLR isoforms, including NLRP1, NLRP2 [13], NLRP3, NLRP6, NLRP7 [14], NLRP12, NLRC4, and NAIP, are key mediators for the activation of inflammasomes in vitro [15].

Inflammasomes are multimeric cell membrane protein complexes that respond to inflammatory responses [16]. Activated inflammasomes release maturated IL‐1β and IL‐18 by promoting the activation of caspase‐1 [17].

### 2.1 Structure

**Figure 1:**
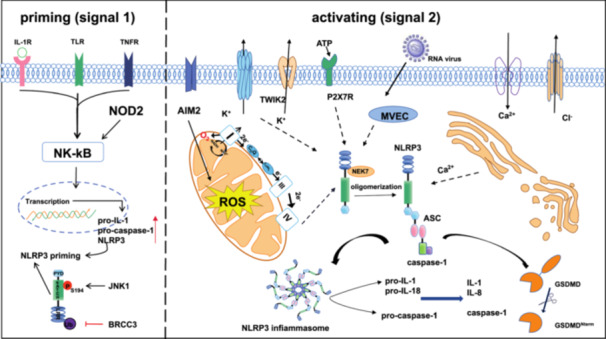
Mechanism of NLRP3 inflammasome activation. Classical NLRP3 inflammasome activation involves two steps. The priming step occurs when inflammatory stimuli are sensed by TLRs, IL‐1Rs and TNFRs to upgrade NLRP3. The activation signal is provided by a wide range of stimuli, including ATP, RNA virus, ion flux AND mitochondrial dysfunction, ROS promote NLRP3 inflammasome activation to release mature IL‐1β, IL‐18 and caspase‐1.

NLRP3 is encoded by the cold‐induced autoinflammatory syndrome 1 gene, detected mainly in the cytoplasm of granulocytes, monocytes, dendritic cells, T and B cells, epithelial cells, and osteoblasts cells [18], which consists of PYD, NACTH, and LRR [19]. The NACHT, essential for NLRP3 self‐oligomerization, belongs to the AAA+ superfamily of ATPase structural domains. Whereas the LRR structural domain is thought to induce self‐suppression by folding back into the NACHT structural domain [20]. NEK7 (NIMA‐related kinase 7) is regarded as a crucial regulator of the NLRP3 inflammasome. It facilitates the assembly and activation of the inflammasome by binding to the LRR domain of NLRP3 in a kinase‐independent manner, which is induced downstream by mitochondrial reactive oxygen species (ROS) [21, 22]. When NLRP3 senses a danger signal, transforming NLRP3 from an inactive double ring cage to an active and more compact disk‐like oligomer [23, 24]. NLRP3 oligomerizes upon itself through homotypic interactions between NACHT structural domains. Oligomerized NLRP3 recruits ASC which is a protein containing a caspase recruitment domain CARD and a heat protein like domain PYD through homotypic PYD‐PYD interactions and forms helical ASC filament formation through PYD‐PYD interactions [25, 26]. Multiple ASC filaments merge into a single macromolecular focal point called an ASC speck (Figure 1). The assembled ASC speck recruit caspase‐1 via CARD‐CARD interactions, by linking the NLRP3 receptor protein to the Caspase‐1 effector protein, the activation of Caspase‐1 is promoted [27]., which then promotes IL‐1β and IL‐18 maturation(Figure 1), inducing inflammation and even cell death known as pyroptosis [28]. It's worth mentioning that in mouse macrophages, recognizing pathogens or injury signals via Toll‐like receptors (TLRs) and activating the downstream NF‐κB pathway are necessary for initiating NLRP3. This activation prompts the cells to produce pro‐inflammatory cytokines, thus preparing for subsequent NLRP3 activation.

### 2.2 Mechanism of NLRP3 Inflammasome Activation

#### 2.2.1 The Priming Process of NLRP3 Inflammasome

NLRP3 Inflammasome activation is strictly regulated. Usually, basal expression levels of NLRP3 are not sufficient to activate the NLRP3 inflammasome. Therefore, most of them are required a two‐step initiation and activation process [29]. The function of initiation is to upregulate NLRP3 inflammasome components, including NLRP3, pro‐caspase‐1, and pro‐IL‐1. Such transcriptional upregulation can be activated by TLRs recognizing microbial components such as lipopolysaccharide (LPS) or endogenous cytokines such as tumor necrosis factor (TNF) after ligand binding to activate the nuclear factor‐κB (NF‐κB) signal pathway (Figure 1), and then through myeloiddifferentiationfactor88(MyD88) and βinterferon TIR structural domain bridging protein (TRIF) signal pathway to upregulate NLRP3 and pro‐IL‐1β [30]. In addition, recent studies have shown that serine/threonine protein kinase 1 (RIPK1), fas‐associated death structural domain protein, and CASP8 receptor interactions serve as another pathway to initiate NLRP3 inflammasome [31]. One study revealed that LPS can induce Interleukin‐1 receptor‐associated kinase 4 phosphorylation, which is a signaling molecule downstream of TLR and MyD88, to acutely initiate NLRP3 inflammasome by IRAK1‐depend on signaling cascade reaction [15]. In addition, protein posttranslational modifications (PTMs) can also initiate NLRP3 inflammasome activation. For instance, TLR4/MyD88 signaling targets NLRP3 deubiquitination by activating deubiquitination enzymes and BRCA1/BRCA2‐containing complex subunit 3 (BRCC3) deubiquitinates the LRR structural domain of NLRP3, to rapidly and non‐transcriptionally trigger NLRP3 inflammasome activation [32]. Besides c‐Jun N‐terminal kinase‐mediated phosphorylation of NLRP3 at S194 is a critical initiation factor to start the transcriptional nondependent initiation of NLRP3 [33]. In conclusion, initiation signaling regulates NLRP3 inflammasome activation through transcription‐dependent and nondependent pathways.

#### 2.2.2 Activates NLRP3 Inflammasome

The NLRP3 is activated by different stimuli after initiation, and multiple molecular and cellular events, including ion flux, mitochondrial dysfunction, and generation of reactive oxygen species, as well as lysosomal destabilization have been demonstrated to trigger its activation [15].

Ionic Flux. K+ efflux is a primary upstream event in activating the NLRP3 inflammasome [34]. In early experiments, it was demonstrated that stimulation of immune cells by PAMPs and DAMPs leads to the massive secretion of ATP via Pannexin, and the increased extracellular ATP, then activates two‐pore domain weak inwardly rectifying K+ channel 2 and purinergic P2X receptor 7 (P2X7R), meanwhile, in macrophages in concert with P2X7R further causes massive K+ efflux to activate the NLRP3 inflammasome [35]. In addition, the binding of never‐in‐mitosis A‐related kinase 7 to NLRP3 promotes molecular complex NLRP3 inflammasome assembly and caspase‐1 activation to regulate the secretion of IL‐1β and IL‐18 [22]. Apart from this, K+ and Cl‐ at decreased concentrations trigger macrophages, monocytes, and neutrophils to activate With‐No‐lysine K (WNK) kinase, which phosphorylates and activates downstream Ste20‐related proline/alanine‐rich kinase (SPAK) and oxidative stress response1 kinases for regulation of NLRP3 inflammasome activation via the WNK‐SPAK pathway [36]. The role of Ca2+ in NLRP3 inflammasome activation remains controversial, Lle G. S, et al. have demonstrated that signals from calcium‐sensitive receptors induce the release of Ca2+ from endoplasmic reticulum stores via inositol trisphosphate 3, thereby increasing cytoplasmic Ca2+ to promote the assembly of inflammasome components; in addition, intracellular Ca2+ signaling via phospholipase C (PLC) stimulation leads to downregulation of intracellular cyclic AMP levels to activate NLRP3 inflammasome and release IL‐1β [37]. In addition to extracellular Ca2+that amplifies this effect [38].

Mitochondrial dysfunction and reactive oxygen species. It is necessary for NLRP3 inflammasome activation which is the production of dysfunctional mitochondrial DNA (mtDNA). The mitochondrial reactive oxygen species (mtROS)/NLRP3 inflammasome signaling pathway leads to the release of fragmented mtDNA and increased production of ROS, which are located around damaged mitochondria and activate NLRP3, releasing the inflammatory factors IL‐1β and IL‐18 and promotes GSDMD/Gsmd cleavage [39]. Initially, Zhou et al., demonstrated that the accumulation of mtROS produced by inhibiting the mitochondrial respiratory chain can activate the NLRP3 inflammasome [40]. Billinghaml K. et al., showed that mitochondrial electron transport chain (ETC) complexes I and II transfer electrons from nicotinamide adenine dinucleotide phosphate (NADH) and succinate to ubiquinone (CoQ), respectively, reducing it to ubiquinol CoQH2, and mitochondrial complex I transfers electrons from NADH to CoQ while pumping protons across the inner mitochondrial membrane to stimulate NLRP3 inflammasome activation via K efflux, meanwhile mitochondrial ETC complexes I, II, III and V inhibitors all prevent NLRP3 inflammasome activation [41]. MtDNA was found to be released into the cytoplasm in an NLRP3‐/mtROS‐dependent manner to support IL‐1β and IL‐18 secretion.

lysosomal destabilization. Unlike ROS produced by mitochondria, the destruction of lysosomes by post‐phagocytosed particles [42], mediates the production of large amounts of ROS by NADPH oxidase and induces NLRP3 inflammasome activation in macrophages [43]. It is seeming that lysosomal disruption appears to be a key step in the activation of the NLRP3 inflammasome by particulate matter, however, the mechanism of its activation is unclear. Previous studies have shown that recognition by NLRP3 through the exposure of human umbilical vein endothelial cells to the lysosomal disruptor Leu‐Leu‐OMe subsequently leads to activation of NLRP3 inflammasome assembly, and then release of mature cytokines [44]. Excessive accumulation of free fatty acids (FFAs) leads to lysosomal disruption and histone B activation. Histone B is released into the cytoplasm, which may trigger mitochondrial dysfunction and increase ROS production [45]. In combination, lysosomal instability from crystalline or granular agonist phagocytosis activates the NLRP3 inflammasome complex and induces caspase‐1 activation.

We need to further investigate the molecular mechanisms of NLRP3 activation under various infectious and inflammatory conditions to fully understand its activation mechanisms.

## 3 Role of NLRP3 Inflammasome in Inflammatory Diseases

**Figure 2:**
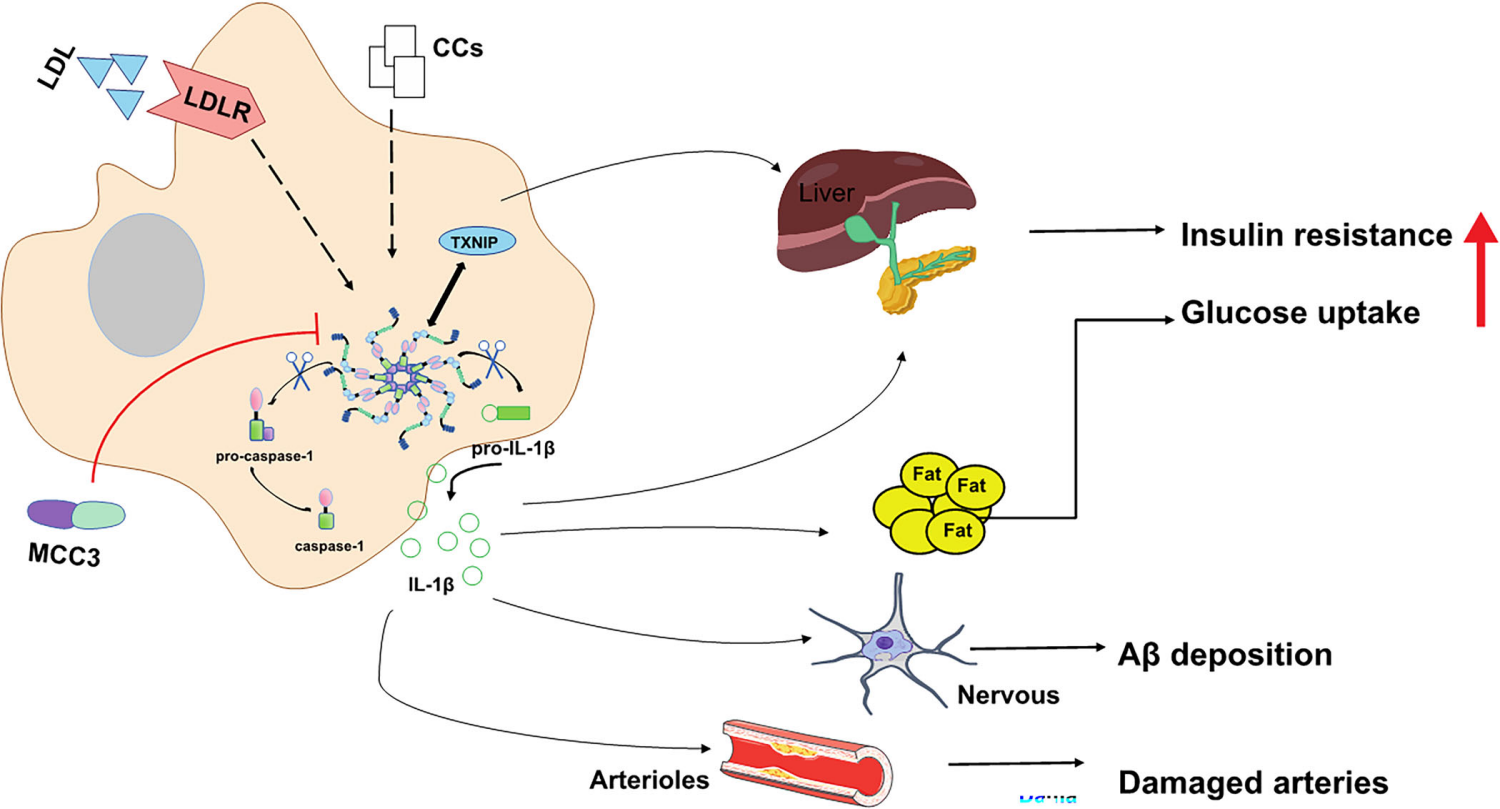
NLRP3 inflammasome in inflammatory disease. LDL, CCS are all reported to regulate activation of the NLRP3 inflammasome and downstream release of IL‐1β leads to insulin resistance, Aβ accumulation in brain and Inflammation of arteries. NLRP3 inflammasome binds to TXNIP to target pancreatic β‐cell apoptosis and inhibit glucose recruitment in peripheral blood, leading to the pathogenesis of diabetes and obesity.

NLRP3 inflammasome play an important role in the activation and regulation of the balance of the innate immunity and inflammatory response of the body. Activated caspase‐1 promotes the processing and secretion of IL‐1β and IL‐18, of which the monocyte is the main secretory cells. IL‐1β promotes the differentiation of Thelper17 (Th17) cells and maintains the production of Th17‐related cytokines. Overexpression of IL‐1β is associated with a variety of diseases (Figure 2). IL‐18 promotes the secretion of Th2 cytokines such as IL‐13 and IL‐4 by mast cells, and T cells which can enhance the Th2 cell‐mediated immune response [46]. Overreaction of the immune response may cause cytokine overactivity and pyroptosis, eventually, resulting in inflammasome diseases including Cryopyrin‐related periodic syndrome, diabetes mellitus, and arteritis, thus understanding the regulatory mechanisms by which NLRP3 inflammasome trigger inflammasome diseases is critical and targeting treatment may show great therapeutic potential.

### 3.1 NLRP3 Inflammasome in Innate Immunity

The inflammasome and mitogen‐activated protein kinases (MAPK) and NF‐κB signaling cascades, initiating the innate and mucosal immune responses. These effects consist mainly of subsequent processes: pro‐inflammatory cytokine release, macrophage hyperactivation with uncontrolled injury, and pyroptosis. During mucosal infection injury, for example, the intestinal epithelial barrier is disrupted, resulting in bacterial invasion of the lamina propria and mucosa. The invading bacteria interact with macrophages, dendritic cells, and neutrophils through innate recognition receptors (e.g., NLRs). Subsequent activation of NLRP3 induces IL‐1β production by colonic lamina propria macrophages, and activated IL‐1βfurther recruits immune cells to the infected tissue, triggering a local mucosal immune response. In contrast, IL‐18 may protect against colitis/colitis‐associated cancers. IL‐18 induces interferon‐γ (IFN‐γ) secretion by Th1 cells and NK cells, which modulates the proliferative and repair response of the intestine during epithelial injury, and in addition, IL‐18 enhances Th1 cell proliferation and host defense against pathogens [47]. Recently, a study showed that exosomes derived through mesenchymal stem cells can inhibit NLRP3 inflammasome and induce macrophage M2b phenotype in macrophages, by decreasing active caspase‐1 to reduce maturation of IL‐1βand IL‐18 and reducing GSDMD pore formation [48]. Similarly, Jiao, Y. et al. revealed that exosomes from multi‐lineage neutrophil sources can promote sepsis‐associated acute lung injury by activating NF‐κB signaling upregulating NLRP3 and inducing M1 macrophage polarization and triggering macrophage pyroptosis [49]. In a view of this, macrophages are increasingly considered a new target for immune homeostasis as a therapeutic target.

### 3.2 NLRP3 Inflammasome in Adaptive Immunity

The NLRP3 inflammasome also plays a vital role in the induction of adaptive immunity, and T helper cells driven by cytokine signaling from dendritic cells and macrophages which are classified as Th1, Th2, Th17, and regulatory T (Treg) cells are important components of the adaptive immune system [47]. It is well known that Treg/Th17 cell balance plays an important role in the progression of rheumatoid arthritis (RA). Park, S. H. et al. showed that NLRP3 can regulate CD4 T cell differentiation to Treg [50], further, using NLRP3‐/‐ mice to construct a model of collagen‐induced arthritis (CIA) found an attenuated inflammatory response and retro‐completion of NLRP3 showed inflammation and a significant exacerbation of Treg/T cell imbalance, which suggests that NLRP3 is involved in the restoration of Treg/Th17 cell homeostasis, and improvement of joint damage [51]. A positive correlation was found between NLRP3 inflammasome expression levels and activity index scores in systemic lupus erythematosus (SLE) patients, that nucleic acid components could activate NLRP3 inflammasome in human monocytes in vitro, which active caspase‐1 was upregulated in monocytes from SLE patients compared to patients with other rheumatic diseases, and that serum titers of anti‐double‐stranded DNA antibodies in monocytes were positively correlated with active caspase‐1 [52]. In addition, Yanghe decoction (a traditional Chinese herbal preparation) could alleviate autoimmune thyroiditis in a rat model by downregulating the NLRP3 inflammasome, decreasing the percentage of Th17, upregulating the percentage of Treg cells, and regulating the imbalance Th17/Treg [53]. These facts suggest that NLRP3 may be involved in the regulatory cellular balance of Treg/T in adaptive immunity.

### 3.3 NLRP3 Inflammasome in Other Inflammatory Diseases

#### 3.3.1 NLRP3 Inflammasome in Cryopyrin‐Related Periodic Syndrome

Mutations in the NLRP3 have been found to cause rare auto‐inflammatory diseases like cryopyrin‐associated periodic syndrome (CAPS), a well‐defined autosomal dominant disorder characterized by generalized, cutaneous, musculoskeletal, and central nervous system inflammation [54]. CAPS has a continuous clinical course with a familial cold auto‐inflammatory syndrome, Mucklewell syndrome, and chronic infantile neurocutaneous‐articular syndrome. It is usually caused by heterozygous NLRP3 gene variants that lead to excessive activation of the inflammasome and subsequent overproduction of IL‐1β [55]. Because of the central role of IL‐1 in the pathogenesis of CAPS, anti‐IL‐1 therapy is recommended for the entire CAPS spectrum [56] (Table 1).

**Table 1:** Investigating the role of NLRP3 inflammasome in autoimmune diseases.

Diseases type	Effect	Pathway	Ref
Colitis	Intestinal local mucosal immune	activate the NLRP3 inflammasome/IL‐β in macrophages	[48]
RA	Chronic arthritis	NLRP3 regulates the differentiation of CD4 T cells into Treg and modulates Treg/Th17	[47]
SLE	Polyarthralgia and arthritis, Raynaud phenomenon, rash on the cheek and other sites, pleuritis and pericarditis etc.	dsDNA/NLRP3/caspase‐1 axis	[49]
CAPS	FCAS, MWS, CINCA	NLRP3 gene mutations	[55]
T1DM	Thirst, overeating, polyuria, and emaciation	Activated NLRP3 inflammasome induces T cell‐specific activation and differentiation, and migration to the pancreas	[61]
Atherosclerosis	Acute inflammation and damage the arterial wall	CCs activate the NLRP3 inflammasome releasing proteinase B, tissue proteinase L or IL‐1	[67]

#### 3.3.2 NLRP3 Inflammasome in Diabetes

NLRP3 inflammasome is a class of metabolic stress receptors, and peritoneal macrophages significantly affect IL‐1β release by inducing its activation and assembly. Elevated levels of IL‐1β are a risk factor in the development of type 2 diabetes mellitus (T2D) and increase insulin resistance [57]. The successful use of targeted IL‐1β therapy for T2D has been further demonstrated in clinical trials, and Anakinra, an IL‐1β receptor antagonist improved blood glucose levels and islet secretion in patients with T2D after 2 weeks of treatment [58]. In addition, The NLRP3 inflammasome plays an important role in regulating T2D by interacting with thioredoxin‐interacting protein (TXNIP), which targets T lymphocyte‐specific mediated apoptosis of pancreatic β‐cells and inhibits glucose recruitment in peripheral blood. Studies have shown that TXNIP ‐/‐ and NLRP3 ‐/‐ mice improved glucose tolerance and insulin sensitivity [59]. Hu C. et al. also revealed that NLRP3 ‐/‐ NOD mice reduced the development of type 1 diabetes mellitus [60]. In conclusion, IL‐1β can induce T cell activation and differentiation, as well as migration of these diabetogenic T cells to the pancreas, which may accelerate the progression from obese individuals to T2D.

#### 3.3.3 NLRP3 Inflammasome in Alzheimer Disease (AD)

AD is a degenerative disease of the central nervous system characterized by the abnormal accumulation of protein aggregates in the form of plaques containing β‐amyloid (Aβ) and the formation of neuronal tangles of neurogenic fibers formed by hyperphosphorylated filaments of the microtubule‐associated protein tau, with clinical manifestations as episodic memory deficits and cognitive impairment. It has been found that the brain is invaded by monocytes/macrophages of peripheral origin that are associated with amyloid plaques and Aβ deposits in the brain, mainly in the phagocytosis of β‐amyloid by microglia into lysosomes, which swell and release histone B, thereby activating the NLRP3 inflammasome and promoting the secretion of IL‐1β in monocytes [61]. Studies have shown increased expression of NLRP3 as well as ASC, caspase‐1, and the cytokines IL‐1β and IL‐18 in cultured monocytes isolated from AD patients [62]. Tejera D, et al. observed that Nlrp3 ‐/‐ mice exhibited significantly less Aβ deposition [63], and treatment of ASC‐deficient APP/PS1 mice with the NLRP3 inflammasome small molecule inhibitor MCC3 improved cognitive function and reduced Aβ accumulation [25, 64]. Furthermore, IL‐1β selectively disrupted hippocampal gamma rhythms in APP/PS1 mice, leading to neuroinflammation that accelerated disease progression [65]. In conclusion, NLRP3 inflammasome as well as IL‐1β are important for the inflammatory response and tissue damage during AD.

#### 3.3.4 NLRP3 Inflammasome in Atherosclerosis

It is well known that inflammation is an important driver of atherosclerosis. Cholesterol crystals (CCs) activate NLRP3 inflammasome and promote the secretion and maturation of IL‐1 family members by caspase‐1‐dependent means. In mice injected intraperitoneally with cholesterol crystals to induce acute inflammation, arterial damage was reduced in mice lacking NLRP3 inflammasome, histone B, histone L, or IL‐1 molecules. In addition, early atherosclerosis and inflammation‐dependent IL‐18 levels were significantly reduced in mice given low‐density lipoprotein receptor‐deficient mice reconstituted with bone marrow transplantation of Nlrp3‐deficient, Asc‐deficient, or Il‐1α/β‐deficient mice and fed a high‐cholesterol diet [66]. This suggests that CCs can activate the NLRP3 inflammasome in mouse and human macrophages. Canasumab, a selective IL‐1β inhibitor, targets inflammation leading to a reduction in cardiovascular events [67]; however, patients have a higher incidence of fatal infections because long‐term inhibition of this important cytokine may compromise host immune defenses [68]. Recently, Vander H. T., et al. demonstrated that the specific NLRP3 small molecule inhibitor, MCC950 (Figure 2), attenuates carotid plaque size in atherosclerosis in apoE ‐/‐ mice by inhibiting NLRP3 inflammasome activation in monocytes and macrophages in mice [69]. These findings may provide new targets for the pathogenesis and treatment of atherosclerosis.

## 4 NLRP3 Inflammasome in Tumors: A Double‐Edged Sword

**Figure 3:**
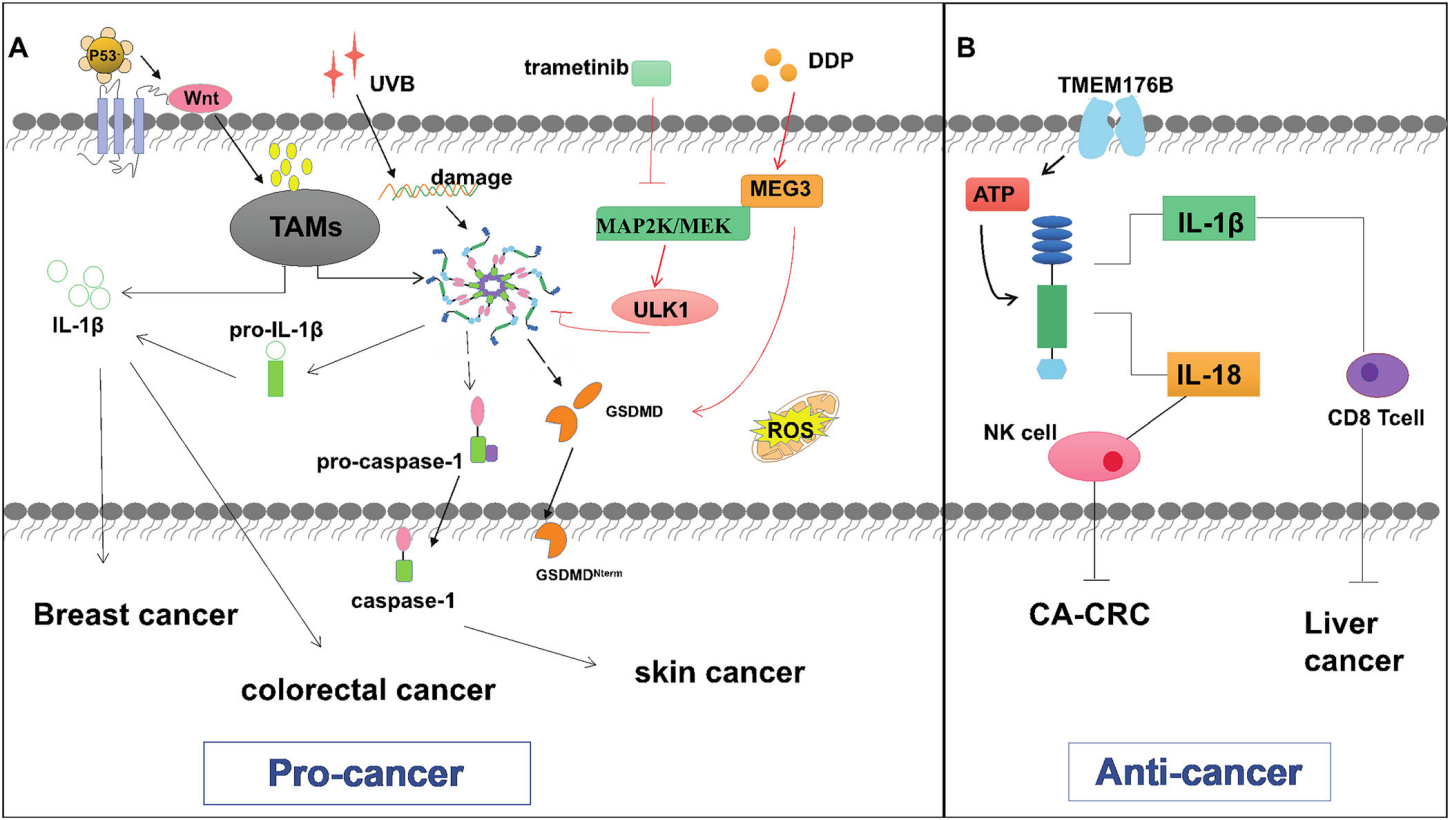
(A) The P53 mutation‐induced WNT signaling pathway mediates the NLRP 3‐IL‐1 β axis in TAMs to promote systemic inflammation and tumor metastasis in the whole body. In primary and metastatic breast tumors, the NLRP3 inflammasome suppresses NK cell activity, releases GSDMD n‐terminal fragments, promoting the maturation and secretion of cytokines IL‐18 and IL‐1 β to, leading to pyroptosis. Overexpressed IL‐1 signaling pathway drives the accumulation of MDSCs and promotes tumors. IL‐1 β also inhibited the antitumor efficacy of the chemotherapeutic agent 5‐FU in CRC. (B) NLRP 3 mediates IL‐18 production and increases NK cell tumoricidal activity, which inhibits metastatic able CA‐CRC tumor cells. NLRP 3 also exhibits a tumoricidal activity against metastatic colon tumor cells in the mouse liver by driving T cell responses via IL‐1β.

The NLRP3 inflammasome acts as a double‐edged sword to influence tumorigenesis and progression by regulating host immunity. An analysis of NLRP3 was reported across 24 cancers, 15 of which showed significantly different expression of NLRP3 inflammasome‐related genes between normal and tumor samples [70]. Normally inflammasome signaling in macrophages largely prevents microbial infections, but aberrant inflammasome signaling promotes chronic inflammation, which can lead to tumorigenesis [71]. NLRP3, inflammasome activation playing a core role in chronic inflammation including immunosuppression, proliferation, angiogenesis, and metastasis, as with breast cancer and cutaneous squamous cell carcinoma (Figure 3); on the contrary, it can also play a tumor suppressive role in relevant colitis and colitis‐associated colorectal cancer (CA‐CRC) [72].

### 4.1 NLRP3 in Tumor Promotion

Macrophages are the most abundant cells in the tumor microenvironment [73]. Tumor‐associated macrophages (TAM) are among the immune cells that infiltrate into tumors and play an important role in tumor lymph angiogenesis and proliferation. The production of IL‐1β and caspase‐1 after NLRP3 activation in TAMs contributes to the formation of an inflammatory tumor microenvironment, which facilitates tumor metastasis [74].

In breast cancer (BC), macrophages comprise 50% of the tumor mass [75]. and TAMs secrete cytokines, chemokines, and enzymes to stimulate cell proliferation, tumor progression and angiogenesis [76]. Wallenstein et al. established a mouse model of breast cancer for analysis which revealed that deletion of p53 induced the WNT signaling pathway and stimulated TAMs to produce IL‐1β, resulting in the promotion of systemic inflammation and tumor metastasis [77]. In addition, high expression of NLRP3 is associated with poor prognosis in BC patients [78], and lack of a functional inflammasome reduces natural killer (NK) cell recruitment and activation in the BC microenvironment [79]. Considering that NLRP3 inflammasome inhibition may be a potential target for the treatment of invasive BC, Yan H. et al. treated invasive BC using cisplatin to induce scorching via activation of the maternally expressed gene 3/NLRP3/caspase‐1/GSDMD pathway in triple‐negative breast cancer (TNBC). Activated caspase‐1 achieves antitumor effects by cleaving the GSDMD and releasing the GSDMD N‐terminal fragment and promoting the maturation and secretion of cytokine IL‐18 and IL‐1β to result in pyroptosis [80]. In addition, Deng R. et al. also used trametinib to inhibit the activation of NLRP3 inflammasome by upregulating unc‐51‐like kinase 1, to trigger mitochondrial autophagy and reduce releasing cytokines, eventually effectively inhibiting bone metastasis [81].

Previous studies have shown that the NLRP3 inflammasome and IL‐1 may also promote skin cancer growth [82]. Drexler et al. observed that the knockout of the Nlpl3 gene or Il‐1 or Caspase‐1 induced fewer skin tumors in mice [77]. Research noted that ultraviolet radiation b‐induced nuclear DNA damage to activate NLRP3, leading to the production of various inflammatory mediators such as IL‐1β, IL‐1α, IL‐6, TNF‐α, and PGE2 [83]. And restored (TAK‐242) as a specific pharmacological inhibitor of TLR4, effectively decreased the expression of NLRP3 and caspase‐1 in skin cells and mice after a single exposure to UVB radiation [84]. In addition, in gastric cancer (GC) through regulation of miR‐223‐3p/NLRP3 axis to induction of NLRP3 inflammasome activation and upregulation of IL‐1β secretion which drives epithelial cell proliferation and tumorigenesis [85]. Furthermore, in KRAS mutant leukemia, activated RAS‐related C3 botulinum toxin substrate 1/ROS/NLRP3/IL‐1β axis causes myeloproliferation and hemocytopenia [86]. Similarly, NLRP3 overexpression in myelodysplastic syndromes directly activates caspase‐1, IL‐1β and focal cell death [87]. NLRP3 can also accelerate head and neck cancer tumor progression by activating cancer stem cells (CSCs) and inducing CSCs to self‐renew [88]. The common denominator affecting the development of inflammation and cancer is IL‐1β levels. In mice, IL‐1β was observed to inhibit IL‐6 and the proliferation and infiltration of myeloid‐derived suppressor cells (MDSC) in TME. Tumor‐associated NLRP3/IL‐1 signaling‐induced MDSC expansion was confirmed, leading to reduced Natural killer and CD8 T cell activity [89]. Previous studies have shown that thymoquinone treatment with mice model could decrease metastatic melanoma by degrading NLRP3 and reducing IL‐1β secretion [90]. These data support the hypothesis that NLRP3 acts as an intrinsic melanoma mechanism driving inflammation and immunosuppression.

NLRP3, caspase‐1, and IL‐1β were highly expressed in human colon cancer cells compared to adjacent normal tissues, and NLRP3 gene expression and protein levels were higher among patients with colorectal cancer grade III than in patients with grade I. This suggests that activation of the NLRP3 inflammasome is associated with disease progression and deterioration, which may involve the epithelial‐mesenchymal transition (EMT) of colon cancer epithelial cells [91]. As NLRP3 may control colorectal cancer (CRC) metastasis by activating EMT [92]. Whereas activation of the NLRP3 inflammasome in CRC by quercetin may reduce the chemotherapeutic potential of the chemotherapeutic agent 5 fluorouracil (5‐FU) [93], suggesting its potential as a therapeutic target. Besides the mentioned tumors, the carcinogenic effects of NLRP3 have been reported in fibrosarcoma [94], lung cancer [95], lymphoma [96], and prostate cancer [97]. In conclusion, these findings suggest a pro‐tumor role for NLRP3 in cancer.

### 4.2 NLRP3 in Tumor Suppression

The NLRP3 inflammasome has also been shown to have antitumor effects. NLRP3 inflammasome signaling in humans is controlled by multiple factors, such as genetic polymorphisms and mutations, that affect gene expression and ultimately lead to its activation. The NLRP3 inflammasome may primarily influence tumor immunity by mediating the actions of tumor‐infiltrating lymphocytes and macrophages [98]. It promotes the activity of effector T cells and natural killer (NK) cells, thereby enhancing their ability to recognize and eliminate tumor cells [99]. Additionally, it regulates the secretion of cytokines within the tumor microenvironment, reduces the expression of pro‐inflammatory cytokines, and ultimately diminishes the growth potential of tumors [100]. In a mouse model of oxidized azomethane‐dextran sodium sulfate (AOM‐DSS)‐induced colon cancer, Nlrp3 knockout mice were more likely to develop colon polyps and were highly sensitive to AOM/DSS‐induced CA‐CRC in a carcinogenic model that exhibited accelerated tumor growth accompanied by colon IL‐18 levels were dramatically reduced. These findings suggest that Nlrp3 can prevent tumor development and that IL‐18 is closely related to colon carcinogenesis. After treatment with AOM‐DSS, Il18 ‐/‐ mice were more apt to produce tumors than controls, while injection of recombinant IL‐18 successfully inhibited disease progression, suggesting that IL‐18 has potential protective and antitumor functions and repair of epithelial damage in CA‐CRC, implying that production of cytokines such as IL ‐18 could be a potential therapeutic candidate for CRC [101]. The latest study showed that Casp1‐/‐ and Asc‐/‐ and Il‐18‐/‐ and Il‐18R1‐/‐ mice also showed increased susceptibility to cancer after AOM/DSS exposure [102]. Nlrp3‐/‐ mice had a higher liver tumor load, Dupaul C J, et al. showed that impairment of inflammasome signaling affects the maturation of hepatic NK cells in tumor‐bearing mice [103]. NLRP3 plays a similar antitumor role in hepatocellular carcinoma patients. In advanced hepatocellular carcinoma patients, the inflammasome expression was reduced or completely lost [104]. Furthermore, the upregulation of NLRP3 by 17 β‐estradiol inhibited the growth of hepatocellular carcinoma cells. Other evidence for instance activation of NLRP3 in kupffer cells inhibited colorectal cancer liver metastasis by releasing IL‐18 and promoting maturation of hepatic NK cells [105]. These all demonstrated a protective role of NLRP3 inflammasome in tumorigenesis. Therefore, IL‐18 signaling downstream of the NLRP3 inflammasome may be critical in preventing colorectal cancer development. What's more, inhibiting transmembrane protein 176B enhances CD8 + T cell‐dependent antitumor immunity in myeloid cells through ATP‐triggered inflammasome activation [106].

## 5 Summary

NLRP3 inflammasome is involved in disease development mainly by promoting the massive release of downstream inflammatory cytokines, generating a pro‐inflammatory microenvironment, inducing malignant transformation, and suppressing local immunity normally provided by NK or T cells, mediating the acute and chronic inflammatory response of the body. Overall, relevant research advances in recent years have greatly deepened our understanding of NLRP3 function and its regulation in host defense, and disease, including tumorigenesis. Despite the clear critical functions of NLRP3 inflammatory in the immune system, their role in cancer remains quite complex and elusive. There is growing evidence that NLRP3 plays a double‐edged role in tumorigenesis and antitumor immunity. these seemingly contradictory functions of NLRP3 inflammasome in tumors may be temporal, contextual, tissue, and cancer specific. There is still much controversy regarding its role in different tumor types, different stages of tumor development, and mechanisms of action in tumor formation, progression, infiltration, and metastasis.

Despite growing evidence that inflammasomes can promote cancer or prevent tumors, many questions remain unanswered. Given the emerging role of NLRP3 in a variety of cancers, NLRP3 inflammasome is emerging as a research topic of interest in the field of the tumor microenvironment, and the potential for their development as important biomarkers for cancer diagnosis or prognosis is quite high. There is considerable potential for the discovery of effective therapies that selectively target NLRP3 inflammasome activation pathways and thereby control tumor formation and progression.

## Author Contributions

Wen‐Liang Li, Ling‐Yan Su, Le‐Lan Gong, Hejiang Zhou, Quan‐Ming Zhao, Ning Xu and Feng‐Chang Huang contributed to the conception and design of the work and drafting of the manuscript. All authors reviewed the content and approved the final version for publication.

## Ethics Statement

The authors have nothing to report.

## Consent

The authors agree to publication in the Journal.

## Conflicts of Interest

The authors declare no conflicts of interest.

## Data Availability

The authors have nothing to report.
